# Tumor suppressive effects of the pleiotropically acting miR-195 in colorectal cancer cells

**DOI:** 10.17179/excli2019-1166

**Published:** 2019-04-09

**Authors:** Zahra Forouzan Jahromi, Arash Javeri, Masoumeh Fakhr Taha

**Affiliations:** 1 Department of Stem Cells and Regenerative Medicine, Institute for Medical Biotechnology, National Institute of Genetic Engineering and Biotechnology (NIGEB), Tehran, Iran

**Keywords:** colorectal cancer, miR-195, EMT, angiogenesis, apoptosis

## Abstract

Downregulation of miR-195 in colorectal cancer tissues has been reported in several studies. We investigated the impact exogenous induction of mature miR-195-5p on some malignant features of human colorectal cancer cells. Caco-2 and SW480 human colon cancer cell lines were transfected with a synthetic miR-195-5p mimic. Exogenous induction of miR-195-5p suppressed multiple mediators of invasion and angiogenesis in colorectal cancer cells and increased the apoptotic cell population in both cell lines. Also, migration of both cell lines was significantly compromised after miR-195 transfection. Our results are indicating a strong tumor suppressive role for miR-195 in human colorectal cancer.

## Introduction

MicroRNAs (miRNAs) are endogenous non-coding small RNAs with a length of about 17-25 nucleotides which act as post-translational regulators of gene expression. In mammals, miRNAs are predicted to control translation of approximately 60 % of protein-coding mRNAs (Friedman et al., 2009[7]). Mature miRNAs repress translation or induce degradation of mRNAs by direct binding to the 3'-untranslated (3´UTR) region of their target mRNAs (Huang et al., 2011[10]). Colorectal cancer (CRC) is the fourth leading cause of cancer deaths worldwide (Siegel et al., 2014[34]). It is well known that development of CRC is a complex process resulting from progressive accumulation of both genetic and epigenetic alterations (Grady and Markowitz, 2002[8]). Abnormal expression of numerous miRNAs has been strongly linked to occurrence and progression of CRC (Dassow and Aigner, 2013[3]). For example, several groups have reported the altered expression of miR-92, miR-21, miR-31, miR-143, miR-145 and miR-143 in CRC (Borralho et al., 2009[2]; Ng et al., 2009[19]; Slaby et al., 2007[35]). Moreover, accumulating evidence has shown abnormal expression of miR-195 in CRC (Sun et al., 2017[36]; Wang et al., 2014[39]; Yang et al., 2015[42]; Zhang et al., 2016[43]). MiR-195 is a member of miR-16/15/195/424/497 family which function either as oncogenic or tumor suppressor miRNAs in various types of human tumors (Katoh, 2014[13]). In CRC, miR-195 acts as a tumor suppressor miRNA and its downregulation has been reported in several studies (Sun et al., 2017[36]; Wang et al., 2014[39]; Yang et al., 2015[42]; Zhang et al., 2016[43]). In the present study, two human CRC cell lines, Caco-2 and SW480, were used to study the impact of exogenous induction of miR-195 using a synthetic form of mature miR-195-5p on several hallmarks of cancer, including apoptotic rate, migration, invasion and angiogenesis.

## Materials and Methods

### Cell culture and miRNA transfection

Human CRC cell lines, Caco-2 and SW480, were purchased from Pasteur Institute of Iran. Both cell lines were cultured in Dulbecco's Modified Eagle's Medium (DMEM) supplemented with 2 mM L-glutamine, 10 % fetal bovine serum (FBS) and 1 % penicillin and streptomycin (all from Gibco, Thermo Fisher Scientific, USA) at 37 ºC in a humidified incubator with 5 % CO_2_. Caco-2 and SW480 cells were seeded in tissue culture plates. At the appropriate density, the cells were transfected with a synthetic miR-195-5p mimic using HiPerFect Transfection Reagent, (all from Qiagen, Hilden, Germany) according to manufacturer's instructions. A mock transfection group for each cell lines was also prepared as the control group. Transfection of the cells was repeated after three days and the cells were collected at the seventh day for downstream applications.

### Quantitative real-time PCR (qPCR) analysis

For analysis of gene expression, total RNA was extracted from the two treatment groups using High Pure RNA Isolation Kit (Roche, Mannheim, Germany). Equal amounts of RNA samples were reverse transcribed to cDNA using RevertAid First Strand cDNA Synthesis Kit (Thermo Fisher Scientific Inc, Waltham, USA), according to the manufacturer's instructions. Experiments were performed using FastStart SYBR^®^ Green Master (Roche Applied Science, Mannheim, Germany) and specific primers for the target genes (Table 1(Tab. 1)) on a Rotor-Gene^TM ^6000 real time analyzer (Corbett Research, Australia). *B2M* (β-2 microglobulin) and *ATCB* (β-actin) were used as reference genes. Comparative analysis of the gene expression data was performed by REST 2009 software (Qiagen).

### Assessment of apoptosis by Annexin V-FITC/PI staining and flow cytometry

The Annexin V-FITC/propidium iodide (PI) staining was performed using Annexin V-FITC/PI Apoptosis Detection Kit (eBioscience, Thermo Fisher Scientific) according to the manufacturer's instructions. Briefly, 1-5×10^5^ cells were collected by trypsinization and stained with Annexin V-FITC and PI.

Stained cells were analyzed by a BD FACSCalibur^TM^ (BD Biosciences, San José CA, USA). Analysis of the apoptosis rate was performed using FlowJo software (Tree Star, Inc., Ashland, OR, USA). In this method Annexin V is labeled with fluorescein isothiocyanate (FITC) which identifies apoptotic cells by binding to phosphatidylserine on the inner leaflet of cell membrane. When paired with an impermeant dead cell stain such as PI, it will be possible to distinguish live, early apoptotic, late apoptotic and necrotic cell populations. 

### Wound healing assay

For wound healing assay, transfected SW480 and Caco-2 cell lines were seeded into 4-well tissue culture plates, and after reaching 80-90 % confluency, the monolayers were scratched with a 1000 μl pipette tip. The cells were washed twice with phosphate-buffered saline (PBS) to remove the detached cells and were incubated in a CO_2 _incubator at 37 ºC for 48 hours under low serum (1 %) condition. Immediately after wound creation and at 48 hours after that, the cells were monitored by an inverted microscope equipped with a digital camera and photos were taken at the mentioned time points. Relative migration distance was calculated by ImageJ 1.51j8 image analysis software (Wayne Rasband, NIH, USA).

### Statistical analyses

Analysis of gene expression was performed by a comparative method using REST 2009 software (Qiagen) based on Pair Wise Fixed Randomisation Test^©^ as described by Pfaffl et al. (2002[24]; Pfaffl, 2001[23]). In this method, the relative expression ratio is calculated from the real-time PCR efficiencies and the crossing point deviation of the two treatment and control samples. Significance of the differences of cell migration distance was assessed by unpaired two-sample *t*-test based on the assumption of obtaining two separate sets of independent and identically distributed samples, one from negative control cells and one from the miR-195-transfected cells. Statistical analysis was performed by GraphPad Prism 5 (GraphPad Software Inc., La Jolla, USA). Four replicates of each treatment group were included in the analyses.

## Results

### MiR-195 downregulated the expression of EMT markers in SW480 and Caco-2 cells

**Figure 1:**
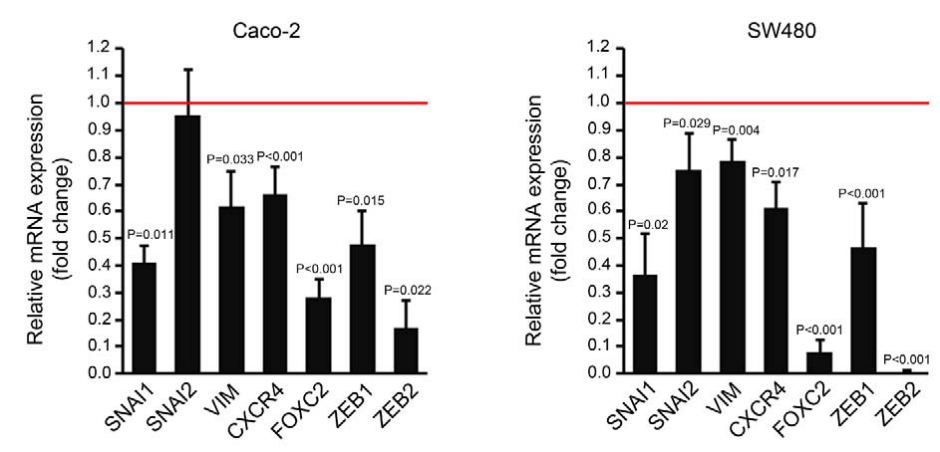
Relative quantification of some EMT genes in Caco-2 and SW480 cells transfected with miR-195 mimic compared to the mock transfected control cells (represented by the red line). Comparative quantification analysis performed by REST 2009 based on Pair Wise Fixed Randomisation Test^©^ (n=4, *P* values generated by REST 2009).

Seven days after the first transfection, the expression of some EMT (epithelial to mesenchymal transition) markers including *SNAI1*, *SNAI2*, *VIM*, *CXCR4*, *FOXC2*, *ZEB1* and *ZEB2* was compared between the cells transfected with miR-195 mimic and the mock transfected cells by qPCR analysis. The expression of almost all the mentioned genes were significantly downregulated in miR-195-transfected Caco-2 and SW480 cells compared to their controls (Figure 1(Fig. 1)). Only *SNAI2* was not affected in Caco-2 cells transfected with miR-195 mimic (Figure 1(Fig. 1)).

### MiR-195 downregulated expression of some angiogenic markers 

**Figure 2:**
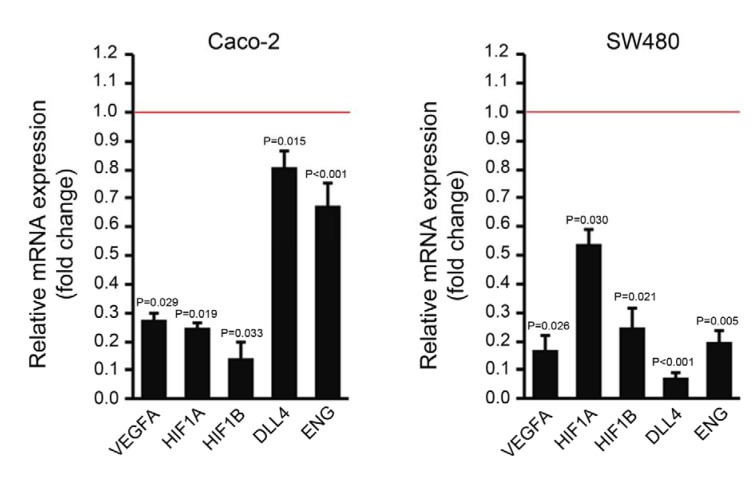
Relative quantification of several angiogenic-related genes in Caco-2 and SW480 cells transfected with miR-195 mimic compared to the mock transfected control cells (represented by the red line). Comparative quantification analysis performed by REST 2009 based on Pair Wise Fixed Randomisation Test^©^ (n=4, *P* values generated by REST 2009).

Seven days after the first transfection, the expression of some angiogenic genes including *VEGFA*,* HIF1A*, *HIF1B*, *DLL4* and *ENG* was compared between the cells transfected with miR-195 mimic and the mock transfected cells by qPCR. The expression levels of all the genes were significantly downregulated in both cell lines after transfection with miR-195 compared to the control group (Figure 2(Fig. 2)).

### MiR-195 induced apoptosis in SW480 and Caco-2 cell lines 

**Figure 3:**
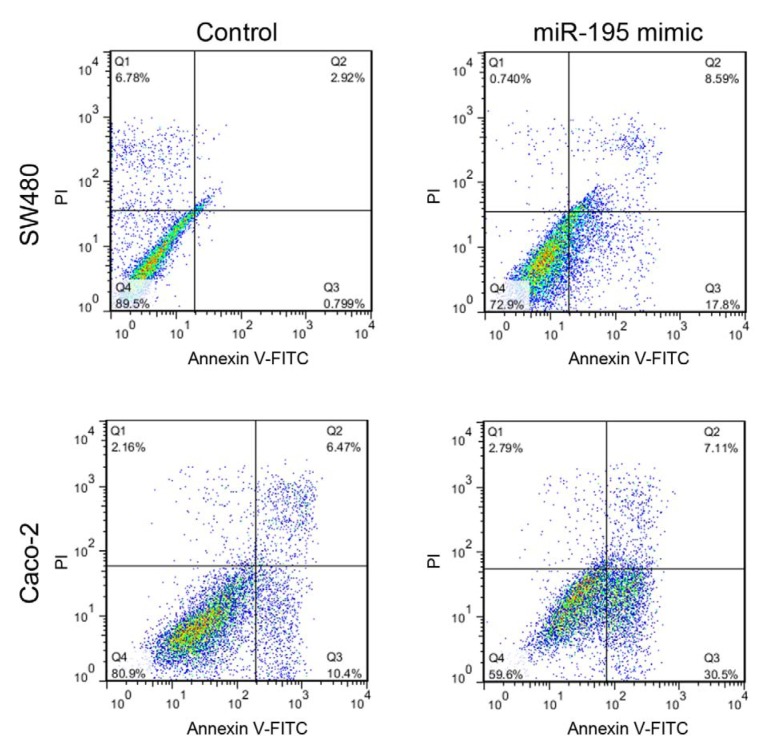
Flow cytometric assessment of the apoptosis rate in SW480 and Caco-2 cells 7 days after miR-195 or mock transfection using Annexin V-FITC/PI staining and flow cytometry. PI: propidium iodide, FITC: fluorescein isothiocyanate. Q1: necrotic cell population, Q2: late apoptotic cells, Q3: early apoptotic cells, Q4: live cells.

Seven days after the first transfection, the SW480 and Caco-2 cell lines were collected and assessed for apoptotic rate by Annexin V-FITC/PI staining and flow cytometry. Transfection of SW480 and Caco-2 cell lines with miR-195 mimic upregulated the rate of apoptotic cells by 17 % and 20 %, respectively (Figure 3(Fig. 3)). 

### Migration of SW480 and Caco-2 cells was compromised by miR-195 

**Figure 4:**
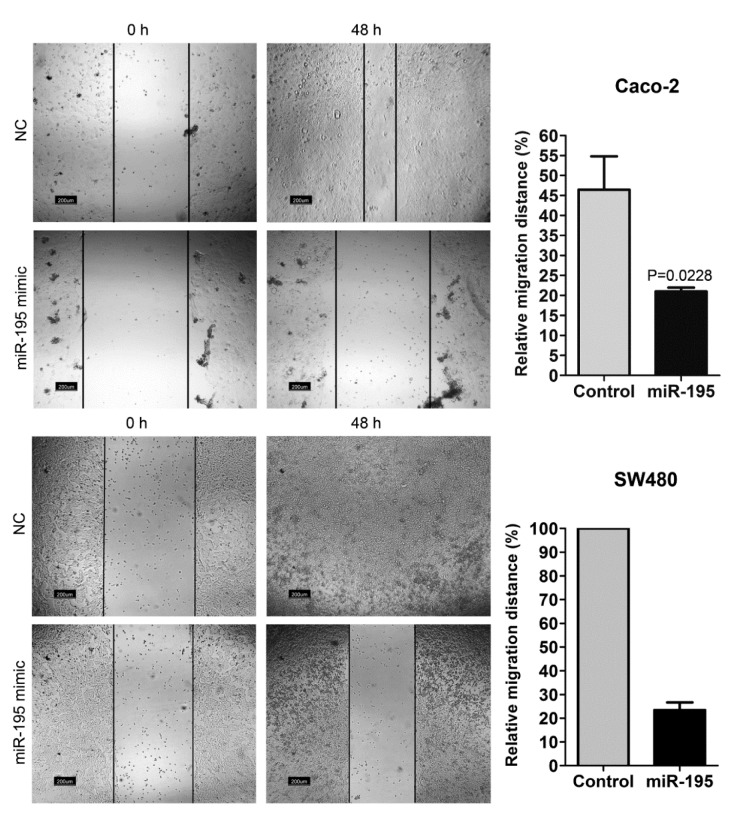
Wound healing assay for assessment of migration rate in Caco-2 and SW480 cells after miR-195 or mock transfection (NC: negative control). Relative migration distance of the cells into denuded area was measured at 0 and 48 h after scratch formation (n=4, unpaired t-test was performed for Caco-2 cells).

Seven days after the first transfection, migration rate of the cells was assessed by wound healing assay. Transfection of both SW480 and Caco-2 cell lines with miR195 mimic resulted in a significant decrease in the migration rate of the cells into denuded area (Figure 4(Fig. 4)). After 48 hours, in SW480 cells the wound was completely closed in the control group while less than 30 % of the wound gap was closed in miR-195 transfected cells. 

## Discussion

EMT is a reprogramming process in which epithelial cells lose their junctions and polarity, reorganize their actin cytoskeleton and acquire the morphological, migratory and invasive phenotypes of mesenchymal cells. These changes are closely linked to cancer formation and progression (Lamouille et al., 2014[15]). EMT-related transcription factors have critical roles in tumorigenesis due to inhibition of apoptosis and senescence. During EMT, epithelial tumor cells are liberated from the surrounding tissue and initiate invasion and metastasis (Puisieux et al., 2014[26]). Also, EMT leads to development of chemotherapy, radiotherapy and molecular targeted therapy resistance in various cancer models (Dave et al., 2012[4]; Holohan et al., 2013[9]; Shibue and Weinberg, 2017[32]; Zheng et al., 2015[44]). Therefore, finding effective strategies which target EMT are of utmost importance in treatment of cancer. In this regard, application of miRNAs capable of targeting EMT-related mediators and mesenchymal genes are of great value. 

MiRNAs play critical roles in regulation of gene expression. A large body of evidence shows the abnormal expression of numerous miRNAs in various types of cancer tissues (Reddy, 2015[29]). In case of CRC, the abnormal expression of numerous miRNAs, including miR-195, has been reported so far (Dassow and Aigner, 2013[3]). MiR-195 acts as a tumor suppressor microRNA and its expression is downregulated in CRC (Sun et al., 2017[36]; Wang et al., 2014[39]; Yang et al., 2015[42]; Zhang et al., 2016[43]). So, in the current study, we aimed to use a synthetic mature miR-195 mimic for overexpression of miR-195-5p in two CRC cell lines exogenously and then assessed how this method affects some malignant features of those CRC cells. We evaluated the impact of this transfection on the expression of *SNAI1*, *SNAI2*, *ZEB1*, *ZEB2*, *VIM*, *CXCR*4 and *FOXC2*. These key transcription factors of EMT potently induce epithelial cell de-differentiation through repression of epithelial genes, including E-cadherin, and upregulation of mesenchymal genes, like N-cadherin (Lamouille et al., 2014[15]; Peinado et al., 2007[22]). Vimentin is a type III intermediate filament protein which is ubiquitously expressed in normal mesenchymal cell. Upregulation of vimentin expression has been reported in various cancer cells, including CRC (Satelli and Li, 2011[31]). In CRC, overexpression of vimentin is correlated with increased migration and invasion of the cells (McInroy and Maatta, 2007[18]), and previous studies collectively indicate the importance of vimentin as a functional biomarker for prognosis of CRC (Alfonso et al., 2005[1]; Satelli and Li, 2011[31]; Shirahata et al., 2009[33]). In our experiment, transfection with miR-195 mimic resulted in downregulation of all the mentioned EMT factors except for *SNAI2* in Caco-2 cells. Therefore exogenous overexpression of miR-195 may serve as an effective suppressor of invasion in CRC cells. Downregulation of EMT markers also support the less migratory capability of the miR-195 transfected cells which was shown by wound healing assay. 

Angiogenesis is a critical step in cancer progression and metastasis and is triggered when the rapidly growing tumor requires an adequate supply of oxygen and nutrients and evacuation of waste metabolic products (Nishida et al., 2006[20]; Quail and Joyce, 2013[27]). Vascular endothelial growth factor-A (VEGFA) is the main regulator of angiogenesis in physiologic and pathologic conditions (Ferrara, 2002[6]). Therefore, the use of different strategies to regulate VEGFA/VEGFR2 signaling pathway can be very beneficial for cancer therapy. So far, several antibodies, aptamers, peptides, and small molecules have been developed to target VEGF signaling in cancer and some of them have been approved by FDA (Niu and Chen, 2010[21]). Recent studies have demonstrated the involvement of a variety of miRNAs in angiogenesis and have suggested the use of either antagomirs or miRNA mimics as a promising strategy for regulation of miRNA levels and targeted angiogenic therapy in various human diseases including cancer (Landskroner-Eiger et al., 2013[16]). To date, a few miRNAs with anti-angiogenic function have been identified in CRC. In this study, we investigated the role of miR-195 in regulation of some angiogenesis factors in two invasive CRC cell lines, SW480 and Caco-2. Transfection of both SW480 and Caco-2 cells with miR-195-5p mimic downregulated expression of *VEGFA* significantly. In addition, several other angiogenesis-related genes, including *HIF1A *(Hypoxia inducible factor-1α), *HIF1B *(Hypoxia inducible factor-1β), *DLL4* (Delta-like 4) and *ENG* (Endoglin or CD105) were downregulated by miR-195. HIF-1 is a heterodimeric transcription factor composed of an oxygen-sensitive subunit, HIF-1α, and a constitutive subunit, HIF-1β (Rankin and Giaccia, 2008[28]). The HIF-1 pathway is the main regulator of cellular response to hypoxia (Krock et al., 2011[14]). HIF-1 pathway activates the expression of several pro-angiogenic genes, including VEGF (Krock et al., 2011[14]). DLL4 is also induced by HIF-1α-VEGF signaling in hypoxic condition (Jubb et al., 2009[12]). Notch ligand DLL4 is expressed in endothelial tip cells of angiogenic sprouts and mediates normal vascular function and organization (Djokovic et al., 2015[5]). Previous studies have demonstrated that Dll4-Notch1 signaling blockade in tumors results in extensive angiogenic sprouting but inhibits tumor growth due to generation of immature and non-functional vasculature (Thurston et al., 2007[37]). Endoglin has crucial role in angiogenesis and it promotes angiogenesis by interaction with vascular endothelial growth factor receptor 2 (VEGFR2) (Tian et al., 2018[38]). It has been shown that in an experimental model of colorectal cancer Endoglin expression possibly has an important role in tumor angiogenesis (Ilhan et al., 2016[11]) and another study on colorectal cancer patients showed a positive correlation of Endoglin overexpression with the presence of angiolymphatic invasion and lymph node metastases (Saad et al., 2004[30]). In the present study, transfection with miR-195 mimic resulted in a significant downregulation in the expression of *VEGFA*, *HIF1A*, *HIF1B*, *DLL4* and *ENG* in both Caco-2 and SW480 lines. Therefore it seems that miR-195 can exert a general suppressive effect on the angiogenic signaling. Wu and colleagues showed that HIF-1α, CXCR4, and VEGF are overexpressed in colon cancer and combined overexpression of any two of these three genes have a significant correlation with lymph node metastasis (Wu et al., 2010[41]). 

We detected downregulation of EMT-related transcription factors including *SNAI1*, *SNAI2*, *ZEB1*, *ZEB2*, *CXCR4* and *FOXC2*, by miR-195 which is in favor of metastasis suppression in CRC. Therefore, overexpression of miR-195 may be used for suppression of both angiogenesis and metastasis of CRC by simultaneous targeting of EMT and angiogenic factors. 

Tumor cells may use different mechanisms to evade apoptosis and acquire apoptosis resistance. Imbalance of pro-apoptotic and anti-apoptotic proteins, downregulation of caspase activity and disruption of death receptor signaling are the main mechanisms which inhibit apoptosis and contribute to carcinogenesis (Wong, 2011[40]). Therefore, targeting any factor or mechanism which is recruited by cancer cells to evade apoptosis may provide an efficient tool to suppress tumorigenesis. As reviewed previously, different sets of miRNAs regulate the intrinsic and extrinsic pathways of apoptosis (Pileczki et al., 2016[25]). MiRNAs function as either oncogenes (oncomiRs) or tumor suppressors and deregulation of them contribute to apoptosis evasion and drug resistance in cancer cells. Current evidence is showing that miR-195 plays a dual role, as an oncogenic or a tumor suppressor, in various types of human tumors (Katoh, 2014[13]). In CRC, miR-195 acts as a tumor suppressor and its downregulation has been found in several studies (Sun et al., 2017[36]; Wang et al., 2014[39]; Yang et al., 2015[42]; Zhang et al., 2016[43]). Herein, we used miR-195 mimic to boost the function of miR-195 in regards to apoptosis. We showed by Annexin V-FITC/ PI staining and flow cytometry analysis that miR-195 mimic promoted cell apoptosis in CRC cell lines, SW480 and Caco-2. Previous studies have shown that the molecular mechanism underlying the apoptotic role of miR-195 in CRC is direct targeting of BCL2 expression (Liu et al., 2010[17]; Yang et al., 2015[42]). However, miR-195 has numerous predicted targets including *CDKN1A*, *CDK1*, *CDK4*, *CDK6*, *CDK8*, *CCND1*, *CCND2*, *CCND4*, *TGFBR3*, *GSK3B* and *FGF2*, which their downregulation can exert pro-apoptotic effects.

In conclusion, miR-195-5p mimic promoted apoptosis and downregulated the expression of several genes involved in EMT and angiogenesis and promoted apoptosis in CRC cell lines. MiR-195-5p is a pleiotropically acting miRNA which has a general suppressive impact on multiple mediators of invasion and angiogenesis in invasive colorectal cancer cells while capable of inducing apoptosis in these cells as well.

## Notes

Arash Javeri and Masoumeh Fakhr Taha (Department of Stem Cells and Regenerative Medicine, Institute for Medical Biotechnology, National Institute of Genetic Engineering and Biotechnology (NIGEB), Pajoohesh Blvd., P.O. Box: 14965-161, Tehran, Iran; Phone: +982144787381, Fax: +982144787399, E-mail: mftaha@nigeb.ac.ir) equally contributed as corresponding authors.

## Acknowledgements

This study was supported by a research grant (No. 940701-I-525) from the National Institute for Genetic Engineering and Biotechnology (NIGEB). All the authors declare no conflict of interest.

## Conflict of interest

The authors declare no conflict of interest.

## Figures and Tables

**Table 1:**
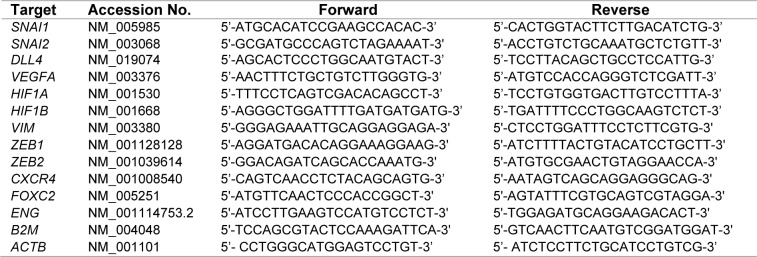
Primers used for qPCR

## References

[1] Alfonso P, Nunez A, Madoz-Gurpide J, Lombardia L, Sanchez L, Casal JI (2005). Proteomic expression analysis of colorectal cancer by two-dimensional differential gel electrophoresis. Proteomics.

[2] Borralho PM, Kren BT, Castro RE, da Silva IB, Steer CJ, Rodrigues CM (2009). MicroRNA-143 reduces viability and increases sensitivity to 5-fluorouracil in HCT116 human colorectal cancer cells. FEBS J.

[3] Dassow H, Aigner A (2013). MicroRNAs (miRNAs) in colorectal cancer: from aberrant expression towards therapy. Curr Pharm Design.

[4] Dave B, Mittal V, Tan NM, Chang JC (2012). Epithelial-mesenchymal transition, cancer stem cells and treatment resistance. Breast Cancer Res.

[5] Djokovic D, Trindade A, Gigante J, Pinho M, Harris AL, Duarte A (2015). Incomplete Dll4/Notch signaling inhibition promotes functional angiogenesis supporting the growth of skin papillomas. BMC Cancer.

[6] Ferrara N (2002). Role of vascular endothelial growth factor in physiologic and pathologic angiogenesis: therapeutic implications. Semin Oncol.

[7] Friedman RC, Farh KK, Burge CB, Bartel DP (2009). Most mammalian mRNAs are conserved targets of microRNAs. Genome Res.

[8] Grady WM, Markowitz SD (2002). Genetic and epigenetic alterations in colon cancer. Annu Rev Genomics Hum Genet.

[9] Holohan C, Van Schaeybroeck S, Longley DB, Johnston PG (2013). Cancer drug resistance: an evolving paradigm. Nature Rev Cancer.

[10] Huang Y, Shen XJ, Zou Q, Wang SP, Tang SM, Zhang GZ (2011). Biological functions of microRNAs: a review. J Physiol Biochem.

[11] Ilhan N, Gungor H, Gul HF, Eroksuz H (2016). Expression of endoglin and vascular endothelial growth factor as prognostic markers in experimental colorectal cancer. Anticancer Res.

[12] Jubb AM, Turley H, Moeller HC, Steers G, Han C, Li JL (2009). Expression of delta-like ligand 4 (Dll4) and markers of hypoxia in colon cancer. Brit J Cancer.

[13] Katoh M (2014). Cardio-miRNAs and onco-miRNAs: circulating miRNA-based diagnostics for non-cancerous and cancerous diseases. Front Cell Dev Biol.

[14] Krock BL, Skuli N, Simon MC (2011). Hypoxia-induced angiogenesis: good and evil. Genes Cancer.

[15] Lamouille S, Xu J, Derynck R (2014). Molecular mechanisms of epithelial-mesenchymal transition. Nature Rev Mol Cell Biol.

[16] Landskroner-Eiger S, Moneke I, Sessa WC (2013). miRNAs as modulators of angiogenesis. Cold Spring Harbor Perspect Med.

[17] Liu L, Chen L, Xu Y, Li R, Du X (2010). microRNA-195 promotes apoptosis and suppresses tumorigenicity of human colorectal cancer cells. Biochem Biophys Res Commun.

[18] McInroy L, Maatta A (2007). Down-regulation of vimentin expression inhibits carcinoma cell migration and adhesion. Biochem Biophys Res Commun.

[19] Ng EK, Chong WW, Jin H, Lam EK, Shin VY, Yu J (2009). Differential expression of microRNAs in plasma of patients with colorectal cancer: a potential marker for colorectal cancer screening. Gut.

[20] Nishida N, Yano H, Nishida T, Kamura T, Kojiro M (2006). Angiogenesis in cancer. Vasc Health Risk Manag.

[21] Niu G, Chen X (2010). Vascular endothelial growth factor as an anti-angiogenic target for cancer therapy. Curr Drug Targets.

[22] Peinado H, Olmeda D, Cano A (2007). Snail, Zeb and bHLH factors in tumour progression: an alliance against the epithelial phenotype?. Nature Rev Cancer.

[23] Pfaffl MW (2001). A new mathematical model for relative quantification in real-time RT-PCR. Nucl Acids Res.

[24] Pfaffl MW, Horgan GW, Dempfle L (2002). Relative expression software tool (REST) for group-wise comparison and statistical analysis of relative expression results in real-time PCR. Nucl Acids Res.

[25] Pileczki V, Cojocneanu-Petric R, Maralani M, Neagoe IB, Sandulescu R (2016). MicroRNAs as regulators of apoptosis mechanisms in cancer. Clujul Medical.

[26] Puisieux A, Brabletz T, Caramel J (2014). Oncogenic roles of EMT-inducing transcription factors. Nature Cell Biol.

[27] Quail DF, Joyce JA (2013). Microenvironmental regulation of tumor progression and metastasis. Nature Med.

[28] Rankin EB, Giaccia AJ (2008). The role of hypoxia-inducible factors in tumorigenesis. Cell Death Diff.

[29] Reddy KB (2015). MicroRNA (miRNA) in cancer. Cancer Cell Int.

[30] Saad RS, Liu YL, Nathan G, Celebrezze J, Medich D, Silverman JF (2004). Endoglin (CD105) and vascular endothelial growth factor as prognostic markers in colorectal cancer. Modern Pathol.

[31] Satelli A, Li S (2011). Vimentin in cancer and its potential as a molecular target for cancer therapy. Cell Mol Life Sci.

[32] Shibue T, Weinberg RA (2017). EMT, CSCs, and drug resistance: the mechanistic link and clinical implications. Nature Rev Clin Oncol.

[33] Shirahata A, Sakata M, Sakuraba K, Goto T, Mizukami H, Saito M (2009). Vimentin methylation as a marker for advanced colorectal carcinoma. Anticancer Res.

[34] Siegel R, Desantis C, Jemal A (2014). Colorectal cancer statistics, 2014. CA: A Cancer Journal for Clinicians.

[35] Slaby O, Svoboda M, Fabian P, Smerdova T, Knoflickova D, Bednarikova M (2007). Altered expression of miR-21, miR-31, miR-143 and miR-145 is related to clinicopathologic features of colorectal cancer. Oncology.

[36] Sun M, Song H, Wang S, Zhang C, Zheng L, Chen F (2017). Integrated analysis identifies microRNA-195 as a suppressor of Hippo-YAP pathway in colorectal cancer. J Hematol Oncol.

[37] Thurston G, Noguera-Troise I, Yancopoulos GD (2007). The Delta paradox: DLL4 blockade leads to more tumour vessels but less tumour growth. Nature Rev Cancer.

[38] Tian H, Huang JJ, Golzio C, Gao X, Hector-Greene M, Katsanis N (2018). Endoglin interacts with VEGFR2 to promote angiogenesis. FASEB J.

[39] Wang L, Qian L, Li X, Yan J (2014). MicroRNA-195 inhibits colorectal cancer cell proliferation, colony-formation and invasion through targeting CARMA3. Mol Med Rep.

[40] Wong RS (2011). Apoptosis in cancer: from pathogenesis to treatment. J Exp Clin Cancer Res.

[41] Wu Y, Jin M, Xu H, Shimin Z, He S, Wang L (2010). Clinicopathologic significance of HIF-1alpha, CXCR4, and VEGF expression in colon cancer. Clin Dev Immunol.

[42] Yang B, Tan Z, Song Y (2015). Study on the molecular regulatory mechanism of MicroRNA-195 in the invasion and metastasis of colorectal carcinoma. Int J Clin Exp Med.

[43] Zhang X, Xu J, Jiang T, Liu G, Wang D, Lu Y (2016). MicroRNA-195 suppresses colorectal cancer cells proliferation via targeting FGF2 and regulating Wnt/beta-catenin pathway. Am J Cancer Res.

[44] Zheng X, Carstens JL, Kim J, Scheible M, Kaye J, Sugimoto H (2015). Epithelial-to-mesenchymal transition is dispensable for metastasis but induces chemoresistance in pancreatic cancer. Nature.

